# Develop Advocacy for Public Health

**DOI:** 10.4103/0970-0218.40870

**Published:** 2008-04

**Authors:** Manmeet Kaur

**Affiliations:** PGIMER School of Public Health, Chandigarh - 160 012, India

“Indian Health Ministry Bags US Award for Health Promotion and Tobacco Control” - this interesting headline appeared two years ago in Indian Health News. Immediately after this, American Cancer Society declared that health ministry of India “is a model to which other nations may aspire.” Persistent efforts of the ministry on national tobacco control legislation and for the leadership Indian delegation provided at the Framework Convention on Tobacco Control(1) is really praiseworthy. Advocacy efforts are building and shaping the social and political climate against tobacco in India. Similar advocacy efforts are also needed for strengthening other disease prevention programs.

First and foremost step in the process of advocacy is identification of problem and there is no denying the fact that health professionals are expert in recognizing the health problem. They are also best at reviewing literature to collect evidence for solving the health problem. However, they often fail to communicate the solution to the public and to the politicians. To be an effective public health professional, sharing of knowledge with public at large is as important as gaining of knowledge. Therefore, health professionals need to advocate for the evidences. World Health Organization defines advocacy for health as “a combination of individual and social actions designed to gain political commitment, policy support, social acceptance, and systems for a particular health goal or program”.(2) It emphasizes responsibility of health professionals as advocates of health at all levels in society. It is though paradoxical that health professionals still relate themselves to treatment of the disease rather than to prevention of the disease, whereas their role as defined in primary health care relates more to disease prevention.

It may be argued that advocacy requires technical know how, evidence based information, identification of stakeholders and opponents. It is believed that rather than devoting their time on advocacy, doctors have bigger things to do; most important of all is ‘saving lives’. Only this can not be the end of the role of a health professional. Political, economic, socio-cultural, behavioral, environmental, and biological factors affect public health. Advocacy aims at making these conditions favorable for health. Therefore, the challenge before all health professionals is to enter into the arena of health advocacy.

Health professionals often remain aloof from advocacy considering that policies and decisions on public health issues are the responsibility of politicians and bureaucrats. Should health professionals not approach political leaders to advocate that health posts should be closer to the community? Of course yes, health professionals have to consider community not as a passive receiver of services but as a stakeholder. And they are not only service providers but are also health advocates. Advocacy and lobbying can not be left to the market forces.

Along with the need of advocacy one needs to understand and recognize that the sustainability and effectiveness of any program can be enhanced by the commitment of policymakers. Seeking such commitment is an important step in planning and launching any health strategy. There are examples from Israel and Canada where nutrition and tobacco control movements have been successful due to effective advocacy and behavior change communication.(3)

It can not be denied that the health professionals collect and share knowledge within their own profession but find it difficult to hold advocacy meetings with the community or with the politicians. They are good at holding formal meetings using technical terms, while advocacy demands informal meetings with stakeholders using simple non technical terms. To make national health programs responsive to the health needs of the community, health professionals require opening dialogue with the political leaders, the policy makers, and the community. Holding advocacy meetings with stakeholders is an important step to create policy environment. Instead of limiting the discussions within the elite professions, civil servants and the government, it is important to involve civil society starting with the informal meetings with the Non-Governmental Organisations to take into account the policy options. Then, the debate can move on to formal meetings with other stakeholders like politicians and bureaucrats.

This is the right time that health professionals take the role of a health advocate to build public opinion. They should also approach media for advocacy using mass media and new technologies, direct political lobbying, social mobilization, and alliance building etc. Chapman characterizes past public health advocacy efforts unplanned. He emphasized that a strategic plan should be adopted after systematic analysis of the public positions of opposing forces.(4)

The issues of equity and public good must be debated using media. Health professionals should clarify the doubts associated with the policy and should lobby for generating positive environment. NGOs represent community and can create support for the policies that can be responsive to the needs of people. Health professionals must share the responsibility of advocating public health cause.
