# Corrigendum

**Published:** 2008-07

In the following article, references were misnumbered. Reference number 4 should be treated as reference number 3, and vice-versa.

Viewpoint

Kaur M., Develop advocacy for public health 2008; 33(2):71-72

Correct references should be as follows:

3. Weinstein Y, Kaluski N. Country profile: Nutrition and education in Israel, Health Promotion: Global Perspectives, 1999 Vol. 2 No.4 Available from: http://www.healthpromotionjournal.com/publications/global/1999-09/1999-09.htm

4. Chapman S. Public health advocacy: A primer. J Epidemiol Comm Health 2004;58:361-5

